# Personal trainers’ health advice in the fitness gym space from a gender perspective

**DOI:** 10.1080/17482631.2020.1794364

**Published:** 2020-10-25

**Authors:** Linn Håman, Helena Yring, Hillevi Prell, Eva-Carin Lindgren

**Affiliations:** a School of Health and Welfare, Halmstad University, Halmstad, Sweden; b Department of Food and Nutrition, and Sport Science, University of Gothenburg, Gothenburg, Sweden

**Keywords:** Diet, exercise, fitness gym, gender, health advice, personal trainer

## Abstract

**Purpose:** This study aimed to describe and problematize the advice on exercise and diet that personal trainers (PTs) provide to their clients from a gender perspective.**Method:** The present study had an explorative design, and the interviews were analysed using an interpretative qualitative approach. Seven focus group discussions were conducted with 19 PTs (aged 23–47 years).**Results:** The findings indicated that the PTs had a *gender-neutral health advice approach* to both women and men, guiding them towards a relaxed attitude to exercise and diet, prioritizing and rationalizing their exercise and diet and eating a natural diet. PTs also had a *gendered health advice approach* as regards women who showed unhealthy exercise and eating behaviours; advising them to eat more and exercise less, to focus on performance rather than appearance and to avoid heavy weightlifting. Some PTs acted evasively and did not give advice to men.**Conclusions:** Both approaches include advice that reflect health as control and health as release. From a gender perspective, PTs health advice both challenged and reproduced the stereotypical male norm in the fitness gym space. However, PTs gendered health advice may create different opportunities for men and women to promote their health and well-being in the fitness gym.

## Introduction

### Health, fitness gym space and gender

In western societies, there is an emphasis on the individual responsibility of one’s health and well-being (Crawford, 1980, 1984; Pelters & Wiljma, 2016). According to this ideology of healthism, health is conceptualized as an achievement as well as self-expression and is inherently linked to self-control (Crawford, 1980, 1984). The ideal body is present in the cultural shaping of health- and body-maintenance practices (e.g., fitness gyms, physical activity and diet); described as fit and firm (Lupton, 1995) and depicted as well-behaved, practising the right healthy habits such as being physically active and consuming a healthy diet signifying self-discipline and control (Crawford, 1984; Lupton, 1995). Fitness gyms have become popular spaces for many people to promote their health and to become fit and achieve an ideal body (cf. Andreasson & Johansson, 2014a; Brogård et al., 2016; Sassatelli, 2012). In this study, the fitness gym as space is a place with invisible, blurred or unspoken and taken for granted boundaries regarding gender (Puwar, 2004). There is some evidence that the gym is stereotypically gendered (Andreasson & Johansson, 2015a; Coen et al., 2018) and the perception is that what men do is usually valued more highly then what women do (cf. Lorber, 1994). From that perspective, women can be perceived as invaders of the fitness gym space when feminine values are invading the male space because the fitness gym is strongly associated with male norms and bodies (cf. Puwar, 2004).

At the fitness gym, men tend to exercise through intensive weight training using free weights (Andreasson & Johansson, 2015a) and consume a strict diet mostly consisting of proteins and in some cases anabolic steroids (Molero et al., 2016). Women, on the other hand, usually take part in group exercise and cardio (Andreasson & Johansson, 2015a; Coen et al., 2018) and tend to use substances for weight loss (Fayet et al., 2012). Excessive interest in dieting may lead to disordered eating (DE) and end with severe clinical eating disorders (Bratland-Sanda & Sundgot-Borgen, 2015; Homan, 2010; Pedersen & Tjørnhøj-Thomsen, 2017). As norms at the gym advocate a large amount of exercise (Rysst, 2010), both DE (e.g., Höglund & Normén, 2002), as well as so-called muscle dysmorphia, have been associated with gym and exercise facilities (Cook et al., 2015; Holland et al., 2014). These unhealthy behaviours and disorders were also shown to exist among PTs and fitness instructors (Bratland-Sanda & Sundgot-Borgen, 2015; Diehl et al., 2016) and tend to be gendered. Disordered eating related to thinness, such as anorexia nervosa and bulimia nervosa, is more frequently reported amongst women (Bratland-Sanda & Sundgot-Borgen, 2015; Homan, 2010). Men, on the other hand, show signs of an obsession related to a striving for muscularity, that, in combination with a skewed conception of the body, could end in muscle dysmorphia (Collis et al., 2016; Diehl et al., 2016; Hughes et al., 2016).

### Health advice, personal trainers and gender

Norms at the gym typically comprise an increased amount of exercise and following a strict diet (Håman et al., 2017; Rysst, 2010) and role models who represent and embody this widely accepted fitness culture are to be found at gyms, some of them working as personal trainers (PTs; Andreasson & Johansson, 2014b; Hutson, 2013). Many people have turned fitness and maintaining a healthy lifestyle into a personal and highly individualized project, consulting PTs to achieve their goals (Maguire, 2001). PTs give advice on exercise, based on client analysis and body testing (cf. Swedish Standard Institute [SIS], 2012). However, PTs have different educational backgrounds and exercise related knowledge (Malek et al., 2002), and sometimes work outside their scope of practice (Anderson et al., 2010). Thus, the delivering of exercise related advice and training programmes could vary considerably among PT professionals, which also report common injuries among clients (Waryasz et al., 2016). Moreover, in the context of exercise, diet play an important part in achieving results, such as gaining muscles or losing weight (Pedersen & Tjørnhøj-Thomsen, 2017; Rysst, 2010). PTs may also offer dietary advice, and some believe it to be an important part of their role (Barnes et al., 2016a; Weissman et al., 2013). In general, PTs seem to be confident in providing dietary advice (Barnes et al., 2016b). However, studies indicate that PTs may possess inadequate basic dietary knowledge (Weissman et al., 2013), extending dietary advice beyond national dietary guidelines (Barnes et al., 2016c, 2017; McKean et al., 2015). PT’s key positions as role models, working in close relationship with clients, may have a profound influence on their clients’ health (Bratland-Sanda & Sundgot-Borgen, 2015; Gavin, 1996). Thus, considering that the PT profession is a growing profession and the great responsibility for clients’ health that PTs possess, it is highly relevant to further study this advice.

Moreover, norms associated to exercise and dietary behaviour appear to be gendered, which consequently may affect PTs’ advice. Sports and exercise, not at least in the fitness gym space, are considered particularly important in the gendering of social realities (cf. Andreasson & Johansson, 2014a, 2015a; Markula & Pringle, 2006). A great body of knowledge demonstrates how gender norms and ideals create dominant symbols and systems that influence social structures, practices, relationships and behaviours (e.g., Butler, 1999; Connell, 2002). The gender norms and expectations derive from social and cultural processes and create gender boundaries, which distinguishes men from women (Connell, 2002). Gender boundaries are visible and invisible, socially and culturally constructed borders that create social differences (Barker-Ruchti et al., 2016; Lamont & Molnàr, 2002). Rules and norms exist which dictate the creation of identities, behaviours and expectations (Connell, 2002). Men and women act according to these rules and norms, which contain concrete perceptions of the relation between men and women and how men and women are supposed to interact with each other in different contexts (Barker-Ruchti et al., 2016; Lamont & Molnàr, 2002). The gender boundaries encompass all parts of society, including the gym space, exercise, and diet, involving a hierarchical power relationship where the man is the norm and is given a higher power position than women (Barker-Ruchti et al., 2016; Lamont & Molnàr, 2002). Masculinity, muscles and strength are higher in social status compared to more female ideals such as slimness and fragility (Barker-Ruchti et al., 2016; Hargreaves, 1994). However, gender norms are not fixed, but fluid (Azzarito & Katzew, 2010), and can be negotiated, transgressed and transformed (Barker-Ruchti et al., 2016). For instance, even if fitness gyms traditionally have been a masculinizing practice, some of the gender norms might have been challenged, negotiated and transgressed since women have entered the gym space both as participants and PTs. Therefore, it is highly relevant to further study what kind of advice that PTs deliver to their clients and how this may be gendered. The present study aims to describe and problematize the advice on exercise and diet that PTs provide to their clients from a gender perspective.

## Methodology

The present study was conducted with an interpretative approach in order to acknowledge PTs’ experience and to allow for their complex and shared realities (Thorne, 2008). The empirical material in the study was drawn from seven focus group discussions (FGDs). FGDs were used since they made it possible to produce data through social interaction when participants create meaning together (Morgan, 1996; Roller & Lavrakas, 2015). The conducted FGDs differed from other forms of group interviews since a researcher moderated the discussions in line with the research purpose and encouraged PTs interaction (Morgan, 1996).

### Sample and procedure

We recruited PTs through purposive sampling (Oliver, 2006). The inclusion criteria were that PTs should represent both women and men and should work at diverse gyms (e.g., as franchise, association form and private company). In the initial phase, we contacted site managers at 13 registered gyms (10 included in gym chains, 2 in association form and 1 private company) for approval in further contacting PTs at the facilities in two cities in Western Sweden. An email with information about the study was then sent to 30 PTs at 10 gyms where the site manager had approved contact. This recruitment did not result in a sufficient number of informants, and an additional email was, therefore, sent to all PTs. Thereafter, a friendly reminder was sent to PTs. Seven FGDs were conducted with a total of 19 PTs (9 women and 10 men). The PTs had varying educational backgrounds (ranging from two weeks to three years) and work experience (see Table I). However, the recruitment process was challenged by PTs working conditions. The ambition was to recruit at least four PTs to each FGD, but in some cases, some of the recruited PTs did not arrive or could not participate.

**Table I.** Focus groups included in the study

Focus-group discussion	Participants (n [gender])	Age range (years)	Work experience as a personal trainer (years)	Gym setting	Duration of focus-group discussion (minutes)
1	4 (3 women, 1 man)	24–43	1,5–4	Franchise	124
2	2 (1 women, 1 man)	23–26	1–5	Franchise	73
3	2 (2 women)	33–40	0,5–1	Franchise	103
4	2 (1 women, 1 man)	31–35	1–12	Franchise	80
5	4 (1 women, 3 men)	23–47	1,5–15	Association form	107
6	3 (1 women, 2 men)	28–47	0,2–5	Private Company	85
7	2 (2 men)	29–35	2,5–6	Franchise	66

Focus group discussions 1 and 5 were conducted as “mini-groups” and contained four PTs in each, and FGD 3 was a “triad” with three PTs (see Roller & Lavrakas, 2015). Focus group discussion 2–4 and 7 only contained two PTs in each group (see Table I). That is fewer participants than traditional focus groups, and therefore they were conducted as Very Small Focus Groups or “dyads” (VSFG; Roller & Lavrakas, 2015; Toner, 2009). Within FGD 1, all PTs had met before; two of them were co-workers at the same gym; the PTs were also co-workers in FGD 5 and 7. In the other FGDs the PTs had not met before.

All FGDs had a moderator and an observer and were conducted in conference rooms at two different universities. A thematic semi-structured interview guide was used, which had been tested by university students in a pilot study. Before the FGDs started, we explained that: a) all statements would remain within the group, b) there were no right or wrong answers, and c) all thoughts and opinions were important. We used follow-up and probing questions throughout the FGDs. After the FGDs, we asked PTs to answer a one-page questionnaire containing background data such as age, sex, education and years working as a PT. The FGDs lasted 66–124 minutes and were audio-recorded and transcribed verbatim.

The study follows the Swedish Research Council’s ethical principles (Vetenskapsrådet, 2017), and is, therefore, in compliance with applicable ethical rules. The PTs were asked to read and sign consent forms before the interviews, where they approved that they understood their rights and the voluntary nature of participation. They were also informed that the collected information would be handled confidentially, and that the FGDs would not be used for anything but research purposes. All PTs have been given pseudonyms to ensure confidentiality.

### Data analysis

**Figure 1.**
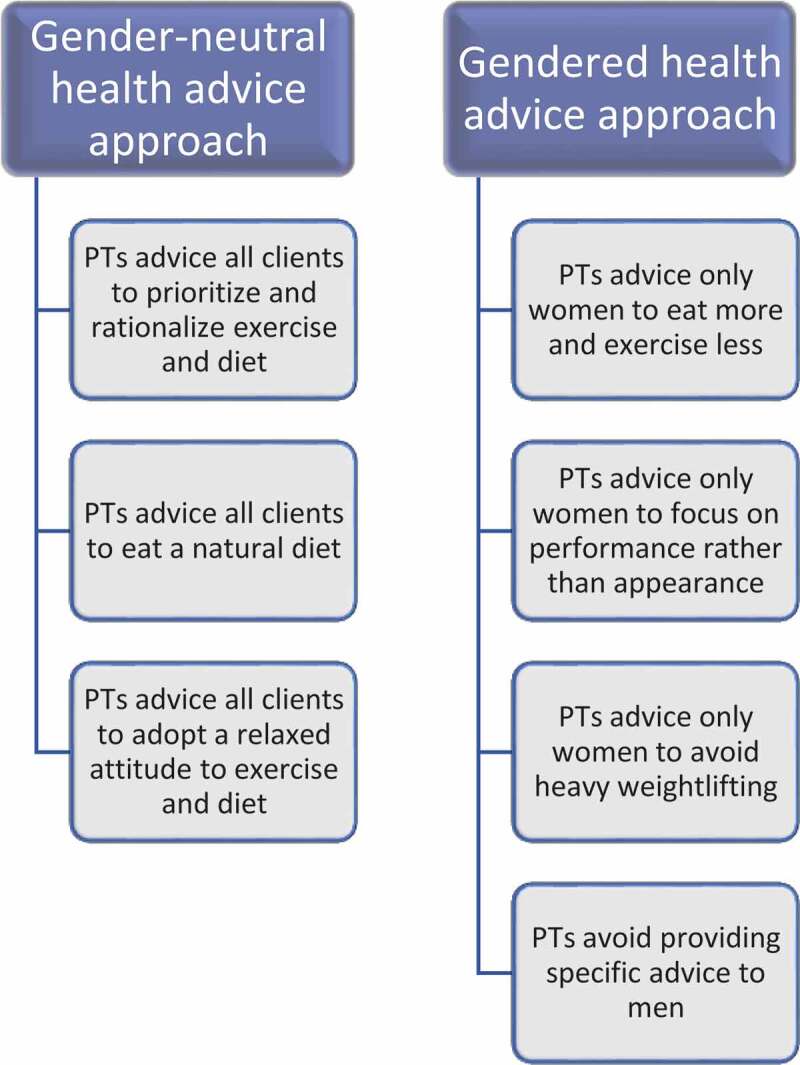
Schematic figure of PTs’ health advice approaches

We employed an interpretative qualitative content analysis (Krippendorff, 2013). The authors (LH, ECL and HY) conducted the major part of the analysis, discussing with the remaining author (HP). We adopted an abductive data analysis approach, which allowed us to engage in a dialectic process of considering data and draw on theory. In the first phase, a qualitative content analysis technique was used (Graneheim & Lundman, 2004), which specifically meant that the interview transcripts were read several times for us to gain an overall impression. Thereafter, meaning units (i.e., segments of conversations or single quotes) were marked that responded to the aim of the study. The marked meaning units were condensed and coded. The codes were compared regarding similarities and differences, then sorted and arranged into 26 tentative categories. The tentative categories were reviewed and discussed with all authors and finally revised and encoded into seven categories covering how PTs in the present study provide health advice: (1) PTs advise all clients to prioritize and rationalize exercise and diet, (2) PTs advise all clients to eat a natural diet, (3) PTs advise all clients to adopt a relaxed attitude to exercise and diet, (4) PTs advise only women to eat more and exercise less, (5) PTs advise only women to focus on performance rather than appearance, (6) PTs advise only women to avoid heavy weight lifting, and (7) PTs avoid providing specific advice to men. In the next interpretative phase, the categories were analysed from a gender perspective in order to strengthen the empirically based conclusions by abstracting the seven categories into two themes: a Gender-neutral health advice approach and a Gendered health advice approach (Figure 1).

## Findings and discussion

PTs’ health advice in terms of gender was formulated as either a *Gender-neutral health advice approach* or a *Gendered health advice approach* (Figure 1). Gendered health advice approach was directed specifically to women only and specific advice directed to men was avoided, whereas gender-neutral health advice was given beyond gender boundaries.

### Gender-neutral health advice approach

The gender-neutral health advice approach refers to the PTs’ general advice to both men and women, in order to promote health by *prioritizing and rationalizing exercise and diet, eating a natural diet* and *adopting a relaxed attitude to exercise and diet.*

#### Personal trainers advise all clients to prioritize and rationalize exercise and diet

The PTs in this study, emphasized that it is important, generally, to prioritize and rationalize exercise and diet to all clients, but for those who want to make lifestyle changes or to lose weight it is fundamental. This advice consists of exercising hard and efficiently while at the gym, but also practical tips on how to find time for and prioritize exercise. For example, clients can set the workout up in the calendar or exercise at home if it is difficult to get to the gym for practical reasons or lack of time. Carolina, one of the PTs said, “Finding time, I would say, not finding excuses// … //there is always time, check how you set your priorities, prioritize exercise by adding it to the calendar like any meeting—then you get it done too” (FGD 2). The advice on diet that PTs gave to clients trying to lose weight and make lifestyle changes was about making the clients pay attention to their dietary habits and break habitual routines. Some of the PTs wrote personal diet schemes or gave their clients a compiled material in order to make it easier for them to follow new routines, which, according to the PTs, can make it easier to succeed with lifestyle changes. PTs often referred to the plate model (Livsmedelsverket, 2017), to describe how the new diet should be composed and described that they also wanted to educate clients regarding a healthy diet.

Mattias: We often talk, purely educationally, about the plate model/ … /to teach them to get away from a habitual behaviour/ … /I believe in making them conscious about how it actually is here and now, and if their objective is to lose weight, this is where you start.

/ … /

Maria: That is what I try to educate them about; you do not need to feel that you have to abstain [from any food]; you can choose a better product and still be content. (FGD 5)

#### Personal trainers advise all clients to eat a natural diet

The PTs in the present study, stressed that a healthy diet was important in order to attain good health. The advice on diet was usually general, but still more explicit than advice concerning exercise. PTs’ dietary advice had its background in the daily energy and nutritional needs of the body and the need of energy in order to be able to exercise, work and function optimally. The PTs discussed that many clients complicated their diet by following specific regimes or by consuming different supplements, protein bars and protein drinks, which was not necessary if they prepared their own food and ate sufficiently. The PTs’ general advice on diet therefore concerned avoiding specific diets, since results can be achieved by consuming ordinary food. In addition, the advice also involved clients exposing themselves to the risk of developing eating disorders if too much focus was on dietary restrictions. In the PT discussions, they pointed out that, if the clients ate a clean diet, as close to its natural form as possible, and avoided snacking and sweets, they would get enough nutrients. Thomas, one of the PTs, said, “[a healthy diet] should contain substances that are as natural// … //as possible” (FGD, 7). Moreover, by looking at the table of contents of products, components that were difficult to recognize or understand, which according to PTs are often less healthy, can be avoided.

Klara: What you find in nature is what we are supposed to eat. Is there a chemistry lesson in the table of contents, then a chemistry lesson is what we should have/ … /it should be healthy and then the question is to find your own way.

Johan: I usually, very simply, say [to clients] to try to eat as much as possible where you can see where the product is from, if you can do that, then the product is hopefully good.

Erik: We talk about clean food, eat chicken fillets, broccoli, brown rice and stuff like that. (FGD 6)

Despite the fact that the PTs gave explicit advice to their clients regarding nutritional content and nutritional needs, some of the PTs expressed an overall restrictive attitude to dietary counselling, with reference to insufficient knowledge and education. Some of them also recommended their clients to contact a trained nutritionist to receive more support and one PT used his partner with an interest in food and cooking to write dietary schedules.

Fredrik: Personally, I do not dare to give [advice].

Stina: No, never!

Fredrik: The more I read about it, the more I understand that you shouldn’t say that much about it.

Anna: No.

Fredrik: It is better to send them to a nutritionist who is more educated than I am. The only advice I can give them is to avoid semi-manufactured products.

Anna: That’s right.

Fredrik: And sweets, and snacks to eat in between meals, but other than that, I do not dare to say more. (FGD 1)

The PTs in this study had a somewhat restrictive attitude towards offering dietary advice as well as an uncertainty concerning appropriate guidelines and nutritional needs. Nevertheless, some of the PTs’ offered explicit advice concerning foods and amount of foods, and some even wrote dietary schedules to their clients, which indicates that they might work outside the recommended scope of practice (cf. SIS, 2012). Lack of basic nutrition knowledge might be one factor that explains PTs’ restrictive attitudes, which has also been shown by a previous study (Weissman et al., 2013). The PTs’ uncertainty might also be explained by the fact that their task assignments do not fall within the domain of personal training (e.g., Gavin, 1996). The dietary advice, in many cases based on PTs’ own preconceived notions and not on scientifically based recommendations, might pose a health risk.

#### Personal trainers advise all clients to adopt a relaxed attitude to exercise and diet

The PTs gave all clients advice to promote their well-being and health by having a relaxed attitude to exercise and diet with the joy of exercise and recovery after exercise as the main focus. According to the participating PTs, this advice aimed to motivate and inspire all clients to reach their goals and adopt lifelong good health where the combination of exercise, diet and recovery was the key to improved health.

Jesper: We know what it takes, and how you get from point A to point B in the best way, and it is diet and exercise, and you need the rest as well, all parts are important. You cannot just stop eating, exercise a lot and skip the recovery. We have a quite common approach [at our fitness gym], a healthy approach, and this is where we start. That is what we are trying to get into our clients’ heads, even if you can tell that they [the clients] have another way of looking at things. They believe that they do not need any rest, but we tell them that you actually do if you want to reach your goals.

Moderator: Mmm

Mattias: I think we have a general, good, healthy approach/ … /For me, a trip from A to B is not just a straight road, but there are many ways of reaching the same goal/ … /many solutions down the road, clearly.

Maria: To get to our clients and bring them back down to earth a little. (FGD 5)

According to the FGDs, the PTs advised their clients to choose an exercise form that they perceived as fun and enjoyable. Thus, they encouraged clients to actively search for a workout form that suited them personally, which did not necessarily have to be at the gym. Furthermore, the PTs described that many clients have a conception of exercise as something that should be painful. As a counterweight, they advised clients to avoid movements that create discomfort or pain and encouraged and guided them to mobility and balance training instead. In relation to this, the PTs pointed out that many clients had a high exercise load, which may lead to clients not getting enough rest and recovery. As a result, the body might be damaged by the strain and become unable to turn exercise into muscles and cardio fitness. Furthermore, the PTs described that clients expressed a desire to get started with everything at once, and to make extensive lifestyle changes in the hope of rapid results. The PTs’ advice was, therefore, not to exercise several times a day, because this could result in injuries and pain and clients dropping out.

Linnea: [We want to] promote a healthy picture and try to make them understand/ … /that if you are going to exercise that much, you have to eat this much.

Moderator: Mmm

Camilla: During consultations, I always talk about how it should be fun; exercise should be fun.

Linnea: Mmm mmm.

Camilla: Don’t exaggerate, don’t set any more goals; [the client wants to] work out six times a week, is that reasonable? We start off a little [more easily] and then you can add more if your main goal is to exercise six times a week, but let’s start with three?/ … /You talk about [what is] normal, that it should be fun and not a constraint.

Linnea: Just enough is actually the best. (FGD 3)

The PTs advised clients not to exclude any food from their diet and to be critical when they were warned about specific foods. Furthermore, they stressed the importance of eating for the soul and enjoyment, since they perceived that clients often expected PTs to give them bans and pointers to what they may or may not eat.

On the one hand, it is possible that PTs’ advice to clients to adopt a more relaxed attitude to exercise and diet as well as eating a natural diet is a consequence of women’s movement into the fitness gym space (cf. Barker-Ruchti et al., 2016). The fitness gyms have historically been a masculinizing practice that cultivated men’s strength and aggression (Andreasson & Johansson, 2014a, 2015a; Molero et al., 2016). On the other hand, the PTs gender-neutral health advice regarding a more relaxed attitude to exercise is given to both male and female clients who often strive for too high exercise frequency. Indeed, this behaviour was shown to be related to an emerging aggressive exercise trend in society (Håman et al., 2017).

### Gendered health advice approach

The gendered health advice approach referred to PTs giving specific advice only to women by recommending them to *eat more and exercise less, focus on performance rather than appearance, avoid heavy weightlifting* and *avoid providing specific advice to men*.

#### Personal trainers advise only women to eat more and exercise less

According to the PTs, women’s exercise and dietary habits were shaped foremost from a striving for thinness and, therefore, they were not eating enough, while following specific diets or completely skipping breakfast. They recommended female clients to eat considerably more, especially women who exercise at a high level, because they often have an energy deficit.

Camilla: In my experience, many [women] eat too little.

Linnea: Yes, [women] are afraid to eat.

Camilla: Yes, I often recommend them to eat more.

Linnea: Mmm

Camilla: What do you eat [PT ask] and then when you start to look [closer], wow, you exercise this much—you need more food. (FGD 3)

According to the PTs, many women choose to do a lot of cardio to burn the food they consume, because of their striving for thinness. This means that many women are regarded to have a problematic exercise behaviour. Therefore, PTs advised them to exercise less, exercise more gently and to reduce cardio exercise and instead focus on weight training and yoga. A recurring pattern in the present study was that PTs tended to express that a high exercise frequency was more problematic for women than for men. When women had a high exercise frequency, it was described as being an unhealthy obsession that, in combination with a low food intake, was given the status of disordered eating and unhealthy exercise.

#### Personal trainers advise only women to focus on performance rather than appearance

Women’s unhealthy behaviours may come from performance anxiety, fixation on appearance and too high demands on themselves, according to the PTs. They discussed that several women do a lot of cardio exercise and only a small amount of lightweight training for their appearance. According to the PTs, women in general were afraid of lifting heavier weights, as they did not want to get big muscles. The PTs experienced a resistance from some women when they tried to make them set more performance-oriented goals. Furthermore, they stated that women to a greater extent than men took part in group exercises, which were combined consecutively or followed by time spent on the treadmill or a cross-trainer. Women exhibiting these exercise habits were usually given advice by PTs because of their thinness. The PTs advised these women to shift their focus from appearance to performance, which is demonstrated in the following excerpt;

Anna: I always try to steer it towards a goal of performance.

Sanna: Mmm

Anna: You made it 5 kilometres on the treadmill, next time we try to run even faster/ … /look, you pushed 15 kilograms more, 20 kg more. You change it towards a more performance-oriented goal. Then, the next time, they can come and say, “I ran, and I lifted this much!”/ … /the goal changes, as they realize that exercise is so much more than getting into shape. It is actually something you practice, something you gain confidence in and something you perform. (FGD 1)

The PTs in this study is trying to identify women who show signs of unhealthy behaviours and to advise them to create realistic goals with a focus on performance instead of appearance as well as to eat more and exercise less. This means balancing the advice to women in order to pursue a healthy lifestyle and not triggering any unhealthy and excessive food and exercise behaviours. Thus, it might be regarded as reasonable that PTs deliver this advice specific to women. A previous study has shown that some women exercising within fitness gyms do that for appearance related reasons, and as such are at an increased risk of developing a negative body image and disordered eating (Prichard & Tiggemann, 2012).

#### Personal trainers advise only women to avoid heavy weightlifting

Some of the PTs discussed that they reacted in a negative way when women chose to exercise hard and lift heavy weights, because they considered women not to be biologically built for exercising hard. The PTs therefore advised women not to exercise that hard, which can be seen in the following excerpt;

Klara: They start to build [muscles], then they burn fat/ … /people stop getting their periods because of low body fat; that’s when it gets sick again.

Erik: Norms and such are completely wrong.

Klara: They are going so damn low [in body weight], however, my colleague is actually pretty good; he tries to explain to the girls that you can’t be that low [in body weight].

Johan: No

Klara: You simply cannot, there is no point; you are a woman. (FGD 6).

PTs might provide specific health advice to female clients who are involved in heavy weightlifting and bodybuilding, because they are regarded to break norms regarding feminine body ideals and thereby becomes unfeminine, acquiring too much muscle mass and having irregular periods. Scholars show that women who engage in bodybuilding are often considered unfeminine (Grogan et al., 2004; McGrath & Chananie-Hill, 2009).

#### Personal trainers avoid providing specific advice to men

Among men, the participating PTs identified certain problematic behaviours, but avoided giving them advice because of biological factors that they believed made men more fit for hard weight training, since they have male sex hormones in contrast to women. One of the PTs said, “Men are able to handle weight training better due to their hormones, and that is why they can have a lower percent of [body] fat before their body stops working” (Jonas, FGD 4). In addition, the PTs discussed that some male gym participants believe that they have sufficient knowledge regarding exercise and do not therefore hire them as a PT. Even if the PTs often see incorrect weightlifting exercises among men in the gym space, they avoid giving advice to clients that are not their own. Moreover, some men were expected to be inherently aggressive due to doping and, therefore, the female PTs did not dare provide specific advice to these clients.

Anna: / … /the personnel are very afraid of dealing with problematic behaviours among people who are doped, since they are very unstable

Sanna: mmm

Anna: / … /to give advice to a guy that looks like this [shows a muscular pose] who stands with these very heavy weights, to tell this guy that he has a problem is not a self-evident thing to do. (FGD 1)

The fact that PTs avoid giving specific exercise advice to male clients can be explained by a dominant gender discourse in society that dictates the creation of certain behaviours and expectations (Connell, 2002). For instance, the PTs referred to biological determinants; the male body being more suited for hard weight training in contrast to the female body which is seen as unsuitable to cope with strenuous exercise (Barker-Ruchti et al., 2016; Coen et al., 2018; Hargreaves, 1994). Men’s strength and aggression contributed to female PTs’ fear and evasive behaviour when considering providing advice to male clients that were regarded as doped. This form of masculinity might contribute to reproduction of hegemonic masculinity and subordinate female PTs’ practices (cf. Connell, 2002). In addition, this hegemonic masculinity in fitness gyms might also disfavour clients in need of health advice. Scholars have stated that many men avoid seeking help since it does not relate to masculinity and male norms (Baum, 2006; Wooldridge & Lemberg, 2016). However, according to the PTs, female clients are to a greater extent than men seen to be at risk of ending up in unhealthy exercise and dietary behaviours due to performance anxiety and high demands. Indeed, perfectionism, EDs and cardio exercise among women have been reported and studied (Bardone-Cone et al., 2017; Holland et al., 2014; Homan, 2010) but these relations have also been found among men in the form of, for example, muscle dysmorphia (Hughes et al., 2016) and anorexia nervosa (Welch et al., 2015). Therefore, it might be contradictory that PTs provide advice to women while men who exhibit similar behaviours do not receive any counselling. When PTs avoid advising men, male clients might end up in pursuing unhealthy and harmful exercise and diet related behaviours (e.g., Collis et al., 2016; Diehl et al., 2016; Hughes et al., 2016). Therefore, it is important that PTs possess sufficient knowledge of healthy and unhealthy behaviours concerning diet and exercise from a gender perspective also to support male clients.

### Concluding reflections

The present study suggested that PTs offered gender-neutral health advice as well as gendered health advice. The gender-neutral health advice approach to both male and female clients challenged the stereotypical male norms, when advocating eating a natural diet and adopting a relaxed attitude to exercise and diet. Historically, the stereotypical male norms at the gym have included a large amount of exercise (e.g. Andreasson & Johansson, 2014a, 2015a; Rysst, 2010) and a strict diet mostly consisting of proteins and in some cases anabolic steroids (Molero et al., 2016). This challenge of stereotypical male norms might be explained by the invading of female clients and female PTs into the male space in the gym, which might blur the boundary between genders (see Puwar, 2004). The fact that also men receive advice regarding a relaxed approach and natural diet might be explained by women having entered the fitness gym space with feminine values (see Puwar, 2004). In addition, the gender-neutral approach consisted of planning and organizing their exercise occasions efficiently to rationalize and to take control over their training (see Crawford, 1984). The advice also comprised adopting routines and diet schemes. To be able to prioritize and rationalize accordingly, clients need to be able to possess self-discipline and self-control. At the same time, PTs advised all clients to let go of some control, for instance, when it comes to which diet to consume and how strict a diet should be, and the amount and intensity of physical exercise. Instead, the PTs encouraged clients to focus on the joy of physical exercise and to be more relaxed in relation to their activities at the gym. This advice appears to be driven by a medical aspect of health e.g., knowledge about nutrition, and the risk of developing compulsive disorders, the body’s need for recovery in order to prevent damage. Thus, PTs advice seems to comprise a tension between health as control and health as release, as described by Crawford (1984).

This tension between health as control and health as the release (Crawford, 1984) could, in some way, also be noticed in the gender health advice. For instance, women-only were advised to create realistic goals with a focus on performance instead of appearance. This advice both reflects health as control and health as a release. Women are encouraged to let go of control of their appearance, such as shifting their focus from the idealized body to goals that comprise performance. This advice also seems to be driven by a medical aspect of health (Crawford, 1984) to prevent unhealthy and excessive food and exercise behaviours. However, performance-related goals also contain to increase control in some way, such as focusing on improving the running distance on the treadmill and/or the weight of dumbbells. Women, in contrast to men, were explicity advised to exercise less, and eat more, i.e., let go of control. Moreover, the fact that also female PTs provided more specific health advice to women than men might be interpreted as female PTs have become insiders in the male space adopting a masculine script (Puwar, 2004). When PTs highlight the fact that women are in need of specific health advice and men are not, it contributes to (re)producing the standard for what is considered to be normal, thus reinforcing traditional gender discourses and gender power relations (Barker-Ruchti et al., 2016). Some of the female PTs were afraid of giving advice to male clients. Evidently, this is one way that a traditional hierarchical power relationship between men and women is (re)produced in the fitness gym space. This might lead to the subordination of female PTs relative male clients and that female PTs are given a lower power position (cf. Connell, 1987). As a consequence, when PTs do not support men with advice to the same extent as to women, it can create unequal conditions for health and well-being.

Finally, findings from this study could be used by gym managers and PT educators to increase their awareness and knowledge about the importance of providing equal opportunities for women and men to improve their health and how to detect unhealthy behaviours. Taking the insights from this study into consideration, more research covering PTs’ competence regarding exercise, diet and gender norms and how they construct knowledge and develop competence for their practice, is needed, since that most certainly have a significant impact on the health of their clients.

### Strengths and limitations

A limitation in this study was the size of some of the FGDs, since most of these were so called very small focus group discussions (VSFG; Toner, 2009). This have been criticized for not generating typical group development stages. However, Toner (2009) has argued that VSFG also can reach these group development stages, which we noted as the discussions proceeded. The ambition was to recruit more PTs to each FGD, but several of the PTs were employed by the hour, and working hours were guided by clients’ appointments, which made the recruitment process difficult. The sample of PTs had varying lengths and levels of PT education, years in the profession and that both men and women participated as well as the FGDs were carried out in two different cities and at gyms in different forms (e.g., gym chains and association), which may increase transferability (cf. Graneheim & Lundman, 2004). The analytical process is another strength, since the themes and categories were discussed and reflected on among the authors in a systematic manner, the first and last author being experienced in the method (cf. Graneheim & Lundman, 2004).
